# Prevention and Treatment of Social Anxiety Disorder in Adolescents: Protocol for a Randomized Controlled Trial of the Online Guided Self-Help Intervention SOPHIE

**DOI:** 10.2196/44346

**Published:** 2023-06-21

**Authors:** Noemi Walder, Thomas Berger, Stefanie J Schmidt

**Affiliations:** 1 Division of Clinical Child and Adolescent Psychology Institute of Psychology University of Bern Bern Switzerland; 2 Division of Clinical Psychology and Psychotherapy Institute of Psychology University of Bern Bern Switzerland

**Keywords:** prevention, treatment, social anxiety disorder, internet-based, adolescent, online intervention, anxiety, psychotherapy, youth, child, comorbid, mental health, social phobia, mobile phone

## Abstract

**Background:**

Social anxiety symptoms are highly prevalent among adolescents and are associated with poor quality of life and low psychosocial functioning. If untreated, social anxiety often persists into adulthood and increases the risk for comorbid disorders. Therefore, early interventions for social anxiety to prevent negative long-term consequences are critical. However, adolescents rarely seek help and often avoid face-to-face psychotherapeutic interventions due to the perceived lack of autonomy and anonymity. Thus, online interventions represent a promising opportunity to reach adolescents who have social anxiety but do not seek help yet.

**Objective:**

This study aims to evaluate the efficacy, moderators, and mediators of an online intervention developed to reduce social anxiety in adolescents.

**Methods:**

A total of 222 adolescents aged 11-17 years with subclinical social anxiety (N=166) or with a diagnosis of social anxiety disorder (N=56) are randomly assigned to the online intervention or a care-as-usual control group. The 8-week guided online intervention is based on the Cognitive Model of Social Phobia and evidence-based online interventions for social anxiety adapted to the specific needs of adolescents. The care-as-usual group will be given access to the online intervention after the follow-up assessment. Participants are assessed at baseline, at 4 and 8 weeks post intervention, and at 3-month follow-up assessment on the primary outcome, that is, social anxiety, on secondary outcomes (eg, level of functioning, fear and avoidance, general anxiety, depression, quality of life, self-esteem, and negative effects of the intervention), on potential moderators (eg, therapy motivation, therapy expectancy, and satisfaction with the intervention), and potential mediators (eg, therapeutic alliance and adherence to the intervention). Data will be analyzed based on an intention-to-treat approach and both groups (intervention and care-as-usual) will be compared at each assessment time point. Furthermore, potential mechanisms of change and generalization of intervention effects on daily life are assessed using an ecological momentary assessment procedure that includes items on maintaining mechanisms of social anxiety, social context, and affect. Participants are prompted 3 times a day during the first 8 weeks of the study and again for 2 weeks following the follow-up assessment.

**Results:**

Recruitment is ongoing; initial results are expected in 2024.

**Conclusions:**

Results are discussed considering the potential of online interventions as a low-threshold prevention and treatment option for adolescents with social anxiety and in light of current advances in dynamic modeling of change processes and mechanisms in early intervention and psychotherapy in adolescents.

**Trial Registration:**

ClinicalTrials.gov NCT04782102; https://clinicaltrials.gov/ct2/show/NCT04782102

**International Registered Report Identifier (IRRID):**

DERR1-10.2196/44346

## Introduction

Adolescence marks a crucial timespan for the development of social anxiety disorder (SAD) with the peak of onset between 13 and 15 years. Prevalence rates in adolescents meeting SAD criteria range from 12% to 36%. Additionally, 16% of adolescents report subclinical levels of social anxiety [1-5]. The burden of disease on adolescents with SAD or subclinical social anxiety is considerable, including poor quality of life, low psychosocial functioning [6-8], and high risk of persistence into adulthood and of developing comorbid mental disorders [9-12]. Promising strategies to prevent or diminish these detrimental effects of SAD include low-threshold early interventions that are delivered online.

Online interventions might be especially appealing to adolescents as [3,13-15] they experience higher stigma regarding seeking and accepting help than adults [16,17], particularly those with social anxiety [13]. Low help-seeking behavior results in few adolescents receiving adequate treatment. This is exacerbated by the lack of available psychotherapeutic services for adolescents worldwide [14,15]. Thereby, online interventions bear the promise to offer adolescents help at a low threshold and with high confidentiality [16].

Meta-analyses have generally supported the efficacy of online interventions in adolescents [17]. These online interventions primarily targeted anxiety and depressive symptoms based on cognitive behavioral therapy (CBT). They have been demonstrated to be superior to passive control conditions (eg, waitlist) and equally efficacious to active control conditions (eg, face-to-face CBT) [18]. Notably, face-to-face interventions targeting several anxiety disorders simultaneously are less efficacious in patients with SAD than other anxiety disorders [19-22]. This emphasizes the need for interventions specifically targeting symptoms of social anxiety such as those proposed in the Clark and Wells or Heimberg model [22,23]. Face-to-face trials imply that the Cognitive Model of social anxiety by Clark and Wells [24] also applies to adolescent samples [25-27]. Online interventions targeting social anxiety in adolescents showed large effects compared to passive (ie, waitlist [28,29]) and active control conditions (ie, online support [30]) but, unlike the face-to-face trials, was equally efficacious compared to a generic CBT program for anxiety [29]. These results are promising but need to be interpreted cautiously as some studies primarily focused on a small sample size with older adolescents with speech anxiety [28] or reported high dropout rates in the study and low adherence rates to the online intervention [29]. Poor engagement with online interventions is a known issue [17]. Human support in the form of guidance (eg, sending summaries and reminders and answering questions) can enhance adherence to and effects of online interventions in adult samples [31,32]. So far, there is no conclusive evidence about adolescents; nevertheless, initial studies suggest that guidance is generally accepted and beneficial for adherence and efficacy [33-36].

Since levels of social anxiety and the incidence of SAD increase during adolescence [37], it is important to detect and treat first signs of SAD as early as possible to prevent the development of a full-blown disorder and address associated high rates of psychosocial impairments early on [8]. Most prevention efforts for anxiety disorders have been delivered as universal programs [38]. Indicated prevention offered to individuals already experiencing elevated, subclinical levels of social anxiety below the diagnostic threshold is still relatively scarce, although may be experienced as more satisfying and less resource-intensive than universal programs [38]. Accordingly, studies suggest that indicated prevention carried out in schools [39] or online [40] can produce small but beneficial effects in adolescent samples. As for interventions addressing social anxiety specifically, an online game to improve social skills [41] and an online cognitive bias modification training [42] were shown to be efficacious in adolescents.

Based on these findings, online interventions may be helpful for adolescents with subclinical social anxiety and with SAD, but our understanding of the mechanisms of change underlying these interventions in adolescent samples is still limited [43].

One method of identifying mechanisms of change is to derive them theory-based and examine them as mediators [43]. Possible mechanisms of change for social anxiety may be the hypothesized maintaining factors of SAD derived from the Cognitive Model (eg, automatic thoughts, self-focused attention, safety behavior, pre-, and postevent processing [24]). In correlational and experimental studies, these factors were positively associated with a higher level of social anxiety [44-47]. However, mediation analysis inherently implies a temporal order of the relationship between the intervention, mediators, and the outcome. Therefore, these variables must be assessed longitudinally ideally at multiple times such as in ecological momentary assessment (EMA) [43,48-50]. Previous intervention studies in adolescents including EMA have mostly assessed global social anxiety, momentary social context, and affect but missed to assess individual maintaining factors [51,52]. As EMA captures events as they occur at the moment, this method thereby minimizes memory bias, incorporates contextual information from participants’ natural environment, and accounts for variability in answering behavior [43,48]. This allows to assess mediators of change and the efficacy in participants’ daily lives and extends the knowledge about intervention benefits to their natural and variable contexts [53]. Eventually, this information may add valuable input to improve interventions in the future.

Further efforts to optimize treatments and outcomes involve the modeling of mental disorders as a network of symptoms to identify central treatment targets [54]. According to the network perspective, a mental disorder is best understood as a complex system of dynamically interacting symptoms [55]. Central symptoms (eg, fear of humiliation) of such a network are supposed to spread quickly by activating other strongly interrelated symptoms, while bridging symptoms (eg, low self-esteem) may link clusters of symptoms. Both central and bridging symptoms may represent important intervention targets as they are supposed to be causally linked to other symptoms of an individual’s network [55-57]. To this point, studies of cross-sectional networks have demonstrated that dysfunctional cognitions function as central symptoms and influence multiple other social anxiety symptoms, especially behavioral components such as avoidance and safety behavior in adolescents [58,59]. Examining social anxiety as networks at symptom level over the course of the intervention could add to this knowledge by exploring whether participants’ network structures change from beginning to end of the intervention, in particular, by identifying relevant core and bridging symptoms as drivers of clinical change, by investigating differences in symptom networks between intervention and control group, and by studying the influence of possible moderators (eg, age, gender, and diagnostic status) on networks.

Overall, first studies suggest that online interventions have positive effects on adolescents with SAD; however, much uncertainty still exists about the efficacy of indicated prevention trials including adolescents with subclinical social anxiety. Moreover, previous research has focused mainly on the efficacy of interventions while neglecting both the mechanisms of change and the symptom-level network structure of SAD.

The primary objective of this study is to examine the efficacy of an online intervention called SOPHIE for adolescents with subclinical social anxiety or SAD compared to a care-as-usual (CAU) control group. Thereby, the name SOPHIE is derived from the German term “SOziale PHobIE” (social phobia). It is hypothesized that SOPHIE will be superior to CAU in reducing social anxiety symptoms post assessment and that the intervention effect will be maintained until follow-up assessment 3 months after intervention completion. Furthermore, it is hypothesized that SOPHIE will also have beneficial effects on secondary outcomes, such as depressive as well as general anxiety symptoms, level of functioning, self-esteem, and quality of life at post assessment and follow-up. It is further hypothesized that intervention effects will be moderated by therapeutic alliance, adherence, treatment motivation, and treatment expectation of the online intervention. Moreover, by measuring social anxiety symptoms in their natural context longitudinally, we will examine if intervention effects translate into everyday life and if the postulated maintaining factors of SAD function as mediators of change. Additionally, we intend to model the network structure of social anxiety symptoms at each time point and observe differences in networks of adolescents (eg, regarding age, gender, and severity of social anxiety).

## Methods

### Study Design

This randomized controlled trial (RCT) investigates the efficacy of the online intervention SOPHIE for adolescents with subclinical social anxiety or a diagnosis of SAD. SOPHIE is compared to a CAU control condition in a 2×2×4 design with a 2-level between-subjects factor (2 experimental conditions: SOPHIE vs CAU), a 2-level between-subjects factor (2 diagnostic conditions: SAD vs subclinical social anxiety), and a 4-level within-subjects factor (repeated measures: baseline, midintervention at week 4, post intervention at week 8, and follow-up 5 months after randomization, ie, 3-months follow-up). All participants are additionally assessed in EMA surveys during the first 8 weeks (from baseline to postassessment) and for 2 weeks after follow-up assessment. Participants in the intervention group have additional process assessments at weeks 2 and 6 during the intervention.

### Ethical Considerations

The trial was registered in ClinicalTrials.gov (NCT04782102) and was approved by the Ethics Committee of the Canton Bern in Switzerland (CEC Bern, Project ID 2020-02501). Any changes to the protocol will be submitted to the Ethics Committee. Adolescents and, if younger than 14 years, their guardians must sign the informed consent form prior to the first assessment. Adolescents and their guardians can withdraw from the study at any time. Withdrawal from the study will not be associated with any disadvantages and is fully documented and explained in the informed consent form. Data collected up to withdrawal will still be used for the planned data analyses. All details about privacy and confidentiality protection are given in *Data Management and Monitoring* subsection. Participants were not compensated for their participation in the study. However, they have the opportunity to participate in a lottery and win Netflix, BookBeat, Spotify, or Apple Music vouchers worth CHF 30 (~US $33).

### Procedures

**Figure 1:**
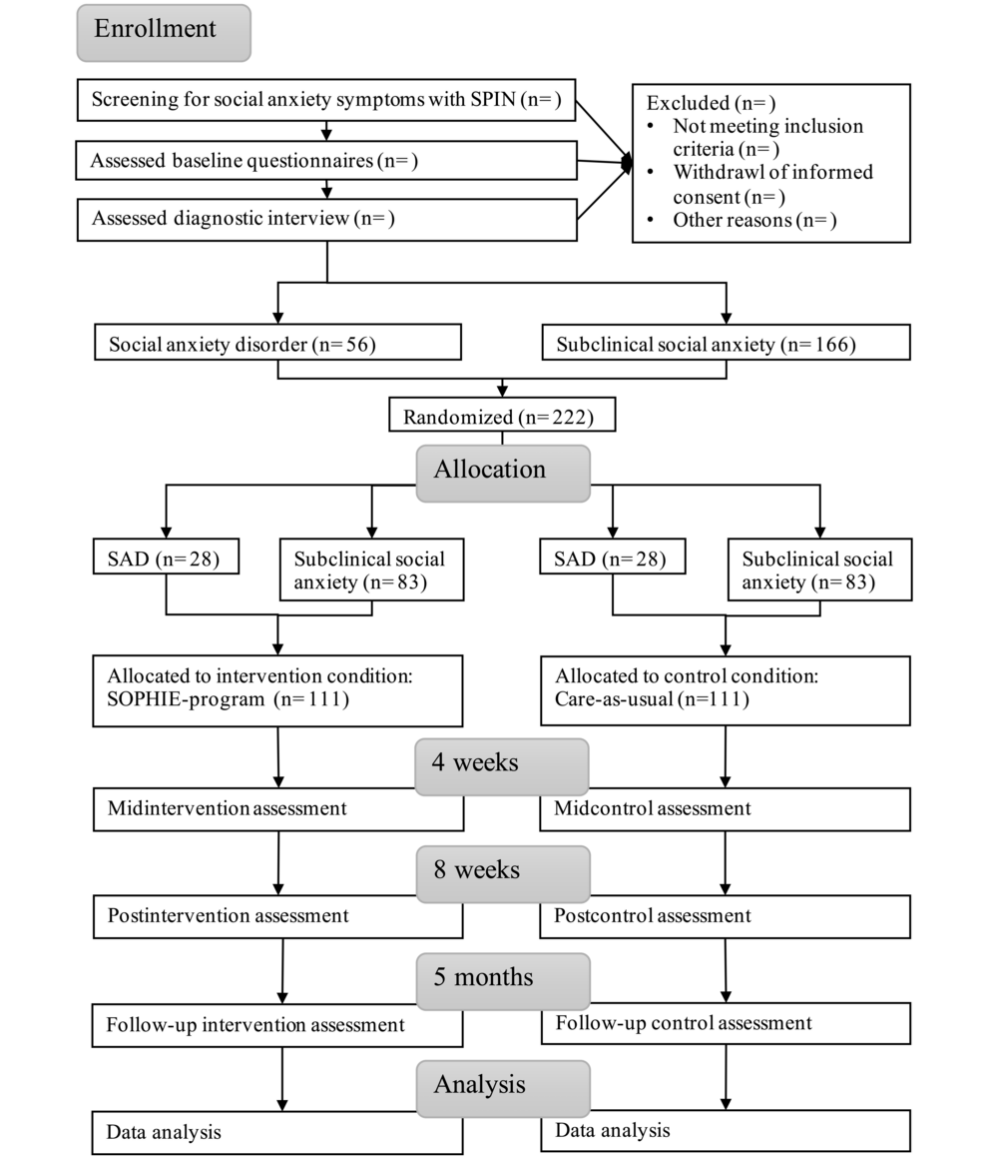
SOPHIE trial flowchart. SAD: social anxiety disorder; SPIN: Social Phobia Inventory.

Adolescents interested to take part in the study leave their email addresses on the study website [60]. They receive the participant study information by email and are asked to provide informed consent or assent (see Figure 1 for study flow). Participants younger than 14 years provide written assent, and their parent or guardian provides written consent. Participants 14 years or older provide consent on their own. Informed consents are accepted both on paper by mail and electronically as scans via email. Once the consent forms have been received, they are printed out and stored in analog form. Afterward, potential participants are screened for eligibility. Those with a score lower than 16 on the Social Phobia Inventory (SPIN) [61,62] are not included in the study but receive immediate access to the online intervention. The cutoff of 16 was chosen in reference to a previous study that showed good discrimination between healthy and subclinical adolescents [63]. Adolescents with a SPIN score of 16 or higher receive the remaining baseline questionnaires. Subsequently, they are interviewed by trained psychology graduate students via phone to ascertain whether they fulfill the diagnostic criteria for SAD or not. Those with a current diagnosis of SAD are assigned to the SAD group, while those with no current and no past SAD diagnosis are assigned to the subclinical social anxiety group. Training and weekly supervision of the graduate students are carried out by the first and last authors.

Participants are assigned to the intervention group or the control group by stratified block randomization to ensure that both groups are balanced regarding the diagnostic status: SAD or subclinical social anxiety. The randomization is carried out after all data collected by the diagnostic interview including diagnoses are entered into the electronic protocol form hosted on Qualtrics (Qualtrics XM) [64]. Randomization was set up before the start of the study by the first author (ie, number of groups, allocation ratio [1:1], and size of blocks). During the study, the allocation list was produced using an automated computer-generated random numbers table and was concealed from the investigators and the participants. After randomization, the intervention group receives immediate access to the online intervention.

During study participation, all participants are reminded twice for each noncompleted questionnaire. For post- and follow-up assessments, an additional reminder is sent out. For EMA surveys, participants are prompted 3 times a day: in the morning before school or work, in the afternoon after school or work, and in the evening with the Smartphone Ecological Momentary Assessment application (SEMA^3^ app) [65].

At post- and follow-up assessment, all participants are contacted again for a telephone interview. Assessors are blinded to the diagnostic status diagnosed at baseline and study condition of the participant, and participants are asked not to mention either of those. CAU group members receive access to the online intervention after they completed all follow-up assessments and the interview.

### Participants, Recruitment, and Eligibility

Participants are recruited in German-speaking countries using advertisements on social media platforms (ie, TikTok and Instagram), self-help forums, and websites (eg, Google ads). Additionally, we share information through partner organizations (eg, school social work, open youth work, and parent counseling), and during school visits. To be included in the study, participants must be between 11 and 17 years of age, understand German, and have access to both a device connected to the internet and a smartphone. In Switzerland, more than 99% of adolescents have their own smartphone and access to the internet [66]. Exclusion criteria are a known diagnosis of autism spectrum disorder, acute suicidality at baseline, and a past diagnosis of SAD for the subclinical group. In case of acute suicidality, we follow a predefined suicidality protocol described in the section “Project and Risk Management.”

### Outcome Measures

#### Primary and Secondary Outcomes

The primary outcome is the severity of social anxiety assessed by the SPIN [67]. The National Institute of Health and Care Excellence guidelines recommend the SPIN as a screening and assessment instrument suited for adolescents [23]. Please see Table 1 for a detailed description and assessment points of the primary and secondary outcomes: social anxiety, social and role functioning, utilization of help, anxiety, depression, quality of life, self-esteem, therapy expectation, therapy motivation, negative effects of the intervention, satisfaction with the intervention, therapeutic alliance, and adherence to the intervention.

**Table 1:** Overview of assessments and assessment points during the SOPHIE study.

Construct		Description of assessment	Timeline of assessment					
			Baseline	Week 2	Midintervention or control at week 4	Week 6	Postintervention or control at week 8	Follow-up at week 20
**Sociodemographic variables**								
	Demographic information	During the first telephone interview, participants are asked about their gender, age, nationality, native language, and living as well as family situation.	✓					
	Socioeconomic status	During the first telephone interview, the *Family Affluence Scale* with 6 items is assessed as an indicator for socioeconomic status and demonstrates good psychometric properties to detect socioeconomic differences in Western European countries [68,69].	✓					
**Primary outcome**								
	Social anxiety	The *Social Phobia Inventory* (SPIN) is a self-report questionnaire assessing 3 dimensions of social anxiety: fear, avoidance, and physiological symptoms. In 17 items, adolescents rate the frequency of each symptom on a 5-point Likert scale from *not at all* (0) to *extremely* (4) ([61], German version by [62]). The SPIN is a reliable self-report measure with good psychometric properties in adolescents [70,71]. Cut-off for subclinical social anxiety symptoms is set at 16 [63].	✓		✓		✓	✓
**Secondary outcomes**								
	Mental disorders	The *structured clinical interview for mental disorders across the life span* is a structured clinical interview to assess the presence of SAD^a^ and comorbid past and present mental disorders according to the Diagnostic and Statistical Manual of Mental Disorders-5 ([72], German Version of Kinder-DIPS; [73]). In this study, the full interview is conducted by phone at baseline. At post- and follow-up assessment, only the section for SAD and sections of previously met diagnoses are assessed.	✓				✓	✓
	Global functioning	The *Global Functioning Social and Role Scale* is a structured interview that is also carried out via phone in this study [74]. Two subscales (social and role) allow for an assessment of the level of functioning (concurrently and in the past year) on a scale ranging from 1 to 10 with 10 representing the highest level.	✓				✓	✓
	Utilization of help	The *Client Socio-Demographic and Service Receipt Inventory* [75,76] is a standardized interview to assess the utilization of help and help seeking behavior. The applied version has already been tested in adolescents [76] and is administered during the postintervention and follow-up telephone interview.					✓	✓
	Fear and avoidance	The *Social Anxiety Scale for Adolescents* measures fear of negative evaluation and social avoidance in adolescents with 18 items answered on a 5-point Likert scale from *not at all* (1) to *all the time* (5) [77]. Validity and reliability were tested in both clinical and nonclinical samples [77,78].	✓		✓		✓	✓
	Anxiety	The *Generalized Anxiety Disorder 7* [79] questionnaire measures self-reported frequency of anxiety symptoms with 7 items answered on a 4-point Likert scale from *not at all* (0) to *nearly every day* (3). The German questionnaire produced good psychometric properties in adolescents [79,80].	✓		✓		✓	✓
	Depression	The *Patient Health Questionnaire-9 for Adolescents* [81] assesses depressive symptomatology with 9 items asking about the frequency of symptoms over the last 2 weeks on a 4-point Likert scale from *not at all* (0) to *nearly every day* (3). The self-report questionnaire has good psychometric properties for depression detection in adolescents [82].	✓		✓		✓	✓
	Quality of life	The KIDSCREEN-10 is used to measure health-related quality of life with 10 items answered on a 5-point Likert scale and has good psychometric properties [83].	✓		✓		✓	✓
	Self-esteem	The *Rosenberg Self-Esteem Scale* [84] assesses self-esteem in 10 items on a Guttman scale. This questionnaire is applicable to different age groups; in this study, the adolescent version is applied.	✓		✓		✓	✓
	Therapy expectancy	The *Credibility or Expectancy Questionnaire* [85] assesses treatment expectancy and credibility with 6 items. There are 2 rating scales, 1 from *not at all* (1) to *very* (9) and another from 0 to 100%. For this study, the wording of the items was adapted to the target group of adolescents.	✓					
	Therapy motivation	The *Motivation for Youth’s Treatment Scale* (MYTS) [86] measures adolescent’s motivation for therapy through a self-report questionnaire. Eight items are rated on a 5-point Likert scale from strongly disagree (1) to strongly agree (5). All adolescents complete this assessment at baseline, only adolescents randomized to the intervention group complete the MYTS at week 2, 4, 6, and 8.	✓	(✓)^b^	(✓)	(✓)	(✓)	
	Negative effects of intervention	The *Inventory for assessment of negative effects in psychotherapy* (German: Inventar zur Erfassung Negativer Effekte in der Psychotherapy, Kinder-INEP; [87,88]) has been adjusted to the online format of the intervention, reducing the questionnaire by 6 items to 15 items. This questionnaire is only answered by adolescents in the intervention group.					(✓)	
	Satisfaction with the intervention	The *ZUF-8* is an 8-item questionnaire for global, unidimensional assessment of patient satisfaction [89]. The questionnaire was adapted accordingly to measure satisfaction with the online intervention. Adolescents randomized to the intervention answer questions on a 4-point Likert scale from 1 to 4.					(✓)	
	Therapeutic alliance	The *Working Alliance Inventory for guided Internet Interventions* (WAI-I; [90]) is a self-report questionnaire that measures therapeutic alliance in online interventions with therapeutic support. The 12 items rated on a 5-point Likert scale from *rarely* (1) to *always* (5) were adapted to adolescents. Only adolescents in the intervention group are assessed with the WAI-I.		✓	✓	✓	✓	
	Adherence to the online intervention	Adherence is operationalized through the extent to which the online intervention is used. The number of finished modules, the number of completed exercises, and the time spent in the online intervention are recorded automatically. As in other studies of online interventions, adherence is calculated with respect to each of these variables and with respect to a composite measure [91].					(✓)	

^a^SAD: social anxiety disorder.

^b^Indicated time points in parentheses reflect assessments only administered in the intervention group.

#### Ecological Momentary Assessment

The same set of EMA questions is provided at all prompts: (1) 10 questions of the *Positive and Negative Affect Schedule for Children* [92] measure positive affect (happy, cheerful, proud, joyful, and lively) and negative affect (sad, scared, miserable, afraid, and mad). The Positive and Negative Affect Schedule for Children has been used in a diary study and showed good psychometric properties in intensive longitudinal designs [93]. (2) The *Social Phobia Weekly Summary Scale* [94] assesses maintaining factors of social anxiety. Six items rated on a 9-point Likert scale from 0 to 8 evaluate the extent of social anxiety, social avoidance, self-focused versus external attention, pre-event, and postevent processing. All items were adapted to reflect the time frame of the EMA measurement (ie, “Since the last prompt”) and the language of the target group. To assess all maintaining mechanisms of social anxiety suggested in the Cognitive Model by Clark and Wells [24] and based on similar existing items [95], we formulated 2 additional items to evaluate automatic thought processing and level of self-confidence in social situations: “In social situations, I have had many negative automatic thoughts running through my head” and “Since the last prompt, I have had little confidence in myself.” Both items are rated on a 9-point Likert scale accordingly. (3) *Momentary social context* is assessed by 4 questions based on similar EMA items from a repository [95]; initially, participants indicate in which social situation they currently are: alone, family, close friends, boyfriend or girlfriend, colleagues, known persons, or strangers. Depending on their answer, they are asked if they would prefer to be alone or with someone (yes or no). Additionally, they indicate if they are currently in contact with others online (yes or no). Finally, they rate their experienced pleasantness in the current situation on a visual analog scale from not at all (0) to extremely (100).

#### Guardian Assessment

Guardians who provide their email addresses on the consent form voluntarily are assessed at baseline, midintervention, postintervention, and follow-up. In all assessments, they rate their child’s social anxiety using the *Parents’ Questionnaire on Social Anxieties in Childhood and Adolescence* (German: Elternfragebogen zu sozialen Ängsten im Kindes und Jugendalter) [96]. In 18 items, statements about the adolescent’s behaviors and fears are rated on a 4-point Likert scale from not at all (0) to very much (3). Furthermore, guardians provide demographic data and answer questions about their child’s treatment motivation and expectations during the baseline assessment. The *Motivation for Youth's Treatment Scale* [86] assesses guardians’ motivation for treatment with 8 items rated on a 5-point Likert scale from do not agree at all (–2) to agree completely (2). The *Credibility or Expectancy Questionnaire—Parent Version* [97] measures guardians’ expectations of the online intervention using 6 items. In total, 4 items are rated on a scale from not at all (1) to very (9), and 2 items on a scale from 0 to 100%.

### Study Group Condition

#### SOPHIE Intervention

The internet-delivered self-help intervention SOPHIE (see Table 2) has a duration of 8 weeks and is based on the Cognitive Model by Clark and Wells [24] adapted to specific needs of adolescents [47] and existing evidence-based online interventions for adults with SAD [98-104]. The first version of the SOPHIE intervention was tested on 46 healthy adolescents. Based on their feedback, we improved the usability and understandability. An introductory module familiarizes adolescents with the intervention. Successively, 6 modules cover the maintaining mechanisms of the Cognitive Model and instruct adolescents to apply the learned strategies in exposure exercises in everyday life. The last module contains a short repetition and summary. The structure of each module includes a review of the previous module, an introduction to the current module’s content, a summary at the end, and a quiz. Content is provided by animated video inputs, short text explanations, and exercises.

To enhance adherence and motivation throughout the intervention, adolescents receive guidance from e-coaches who provide weekly personalized support via a secured text-based messaging system. They summarize completed exercises, provide a preview of upcoming modules, send reminders to use the intervention, answer questions, and motivate participants by relating exercises to their personal goals. e-Coaches are psychology graduate students who were trained by the first or last author in offering guidance. Before sending, each guidance message is additionally peer-reviewed by another e-coach.

**Table 2:** Description of all modules of the SOPHIE program^a^.

Module	Image	Content
(1) Introduction	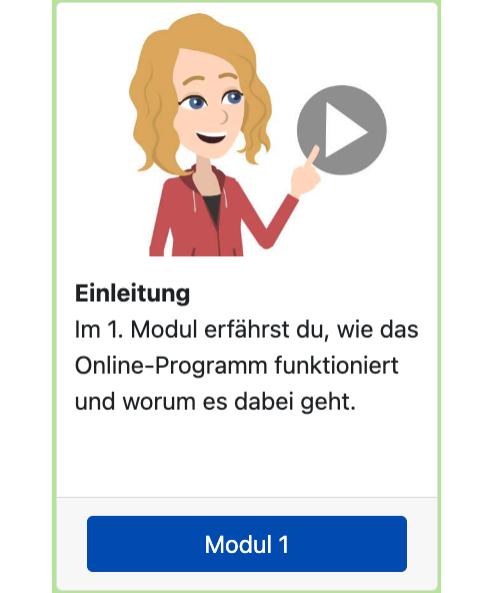	A user guide explains the structure and different elements of the online intervention. Participants are introduced to the 2 protagonists, Sophie and Leon, and can choose their avatar. They learn what social anxiety is, in which situations they may arise, and *set their personal goals* for the online intervention. Additionally, they are introduced to a progressive muscle relaxation exercise and are instructed to do this exercise daily.
(2) Emergence and maintenance of social anxiety	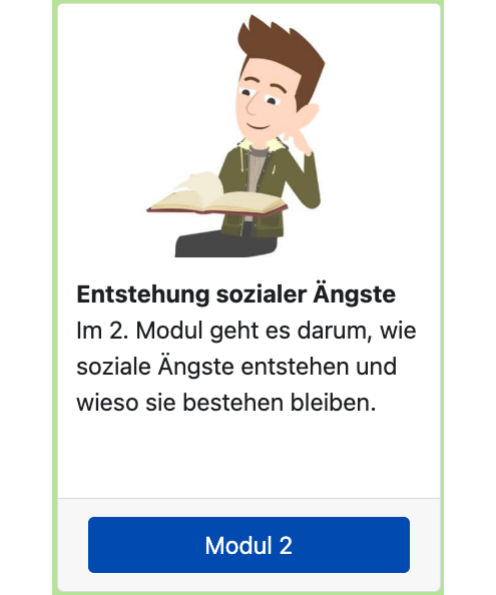	Participants learn about *etiological and maintaining factors of social anxiety*. In an exercise, they write down their personal anxiety cycle. For the following week, they are instructed to observe their social anxiety symptoms and enter their observations in their logbook.
(3) Automatic thoughts	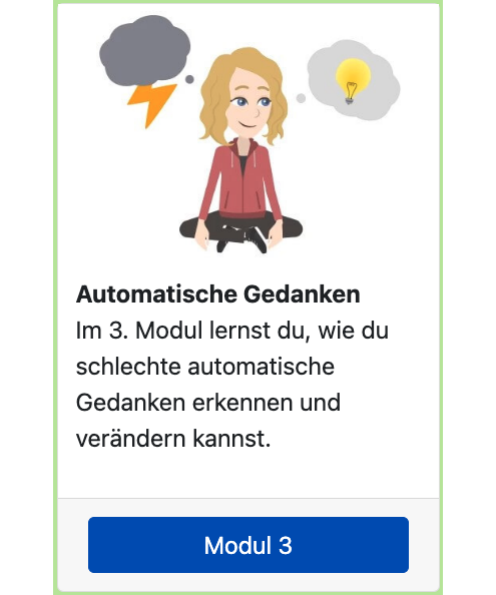	Participants are introduced to *automatic thoughts* and their importance in maintaining social anxiety. In 2 exercises, they are instructed on how to recognize and modify their negative automatic thoughts. An additional relaxation exercise “safe place” is introduced to support their daily relaxation practice.
(4) Self-image	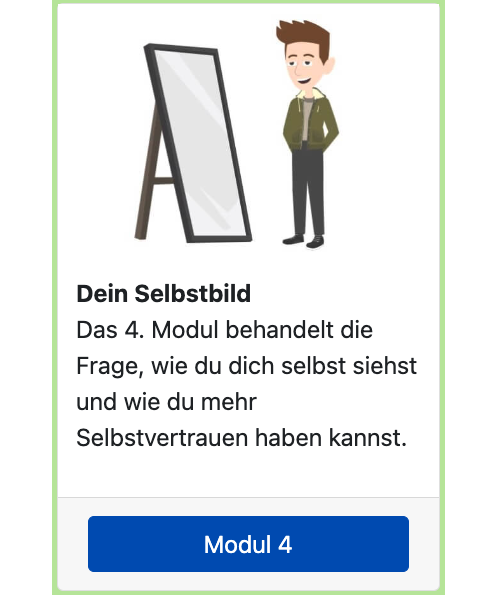	Participants learn about *self and other perspective* of a person, reasons for a positive self-image as well as possible consequences of a negative self-image. During an exercise, they are instructed to reflect on their assumptions of themselves, to recognize both negative and positive sides thereof, and to find a good balance. Participants learn 3 strategies to positively improve their self-image.
(5) Self-focused attention	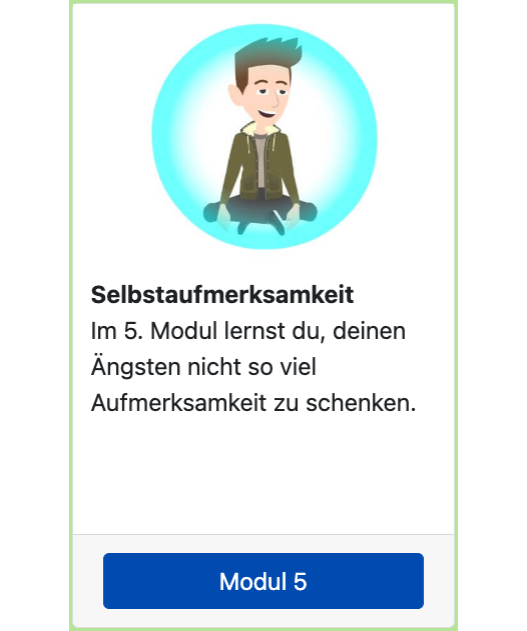	Participants discover *self-focused attention* and the reinforcing impact it has on social anxiety. In an exercise, they train to shift their attention. In front of a virtual audience, they must tell a story and are instructed to focus their attention on the situation instead of themselves. They are introduced to a new relaxation exercise “relaxing world” to complement their relaxation practice.
(6) Reality tests I	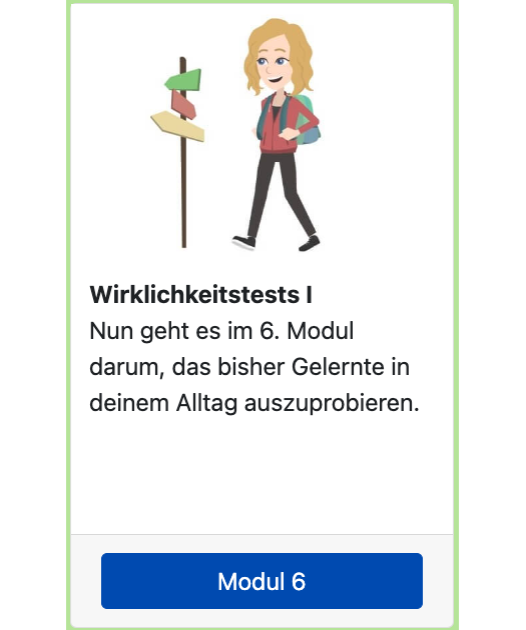	Participants are introduced to reality tests (*exposure exercises*). To prepare for their own reality tests, participants establish their fear hierarchy. In the upcoming week, they conduct their reality tests and record the level of anxiety before and after.
(7) Reality tests II	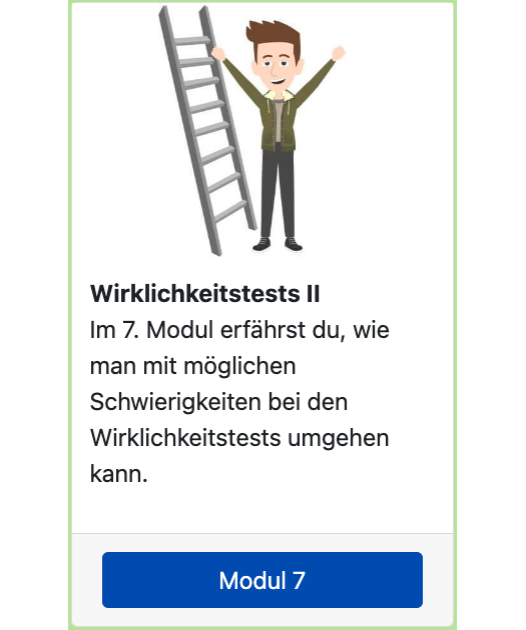	Participants learn about strategies to deal with possible difficulties in reality tasks. Further, they are introduced to *socially competent behavior*. Thereby, examples of competent behavior are contrasted with examples of insecure behavior in social situations. Participants are instructed to continue with their reality tests.
(8) Repetition	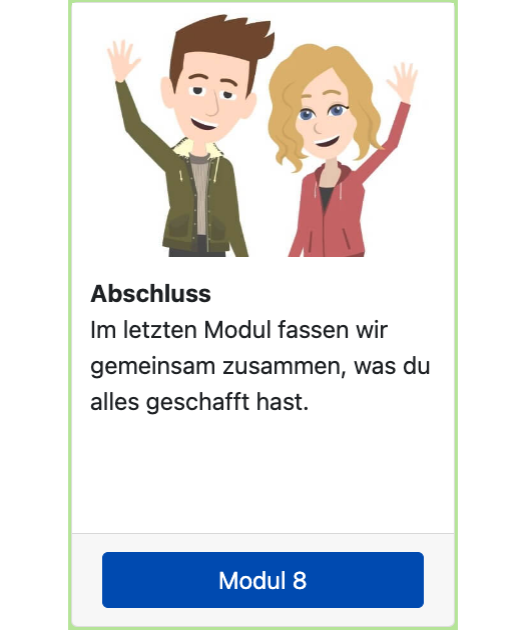	The last module summarizes the content of the online intervention and participants reflect on their personal goals. They record a video of themselves in which they explain what social anxiety is, how they experience it, and how they learnt to cope with it. Later, they can replay this video to remind themselves of their progress.

^a^Displayed pictures were generated with the video program Vyond [105].

#### Care-as-Usual Group

Participants randomized to the CAU group are only contacted for assessments and receive access to the online intervention after follow-up. They are free to take advantage of any other health service. To account for additional help, the use of assistance is assessed post intervention and follow-up (details in Table 1).

### Project and Risk Management

In weekly monitoring procedures, project implementation, clinical (eg, study withdrawals and adverse events), and research integrity (eg, blindness, data safety, important harms, or unintended effects) are ensured. Potential risks to participants are minimal. However, there is a potential inconvenience as filling out the questionnaires and the online intervention requires time.

In the case of suicidality (assessed at every assessment point by item 9 of the Patient Health Questionnaire [81]), a predefined suicidality protocol is applied; participants immediately receive an email with numbers for psychiatric services. Additionally, a member of the study team contacts the participant on the following working day to clarify acute suicidality. If acute suicidality is present, we offer additional help for instance by providing contact details on mental health services near to the adolescent’s home, if necessary, we support the referral to an appropriate service and decide on the continuation in the study. All participants must provide a safety contact in case that we cannot reach the adolescent who triggered the suicidality protocol.

Adverse events, all untoward events in a participant, including events not necessarily caused by or related to the online intervention, are monitored from randomization to follow-up 5 months after randomization. All adverse events are reported to the principal investigator, the ethics committee, and are documented in the participant’s case report form.

### Data Management and Monitoring

All data are collected electronically except for informed consent and case report forms. Electronic data from all servers are extracted monthly and saved on a secure server of the University of Bern. All data are deidentified by removing names, email addresses, and location information, and are only accessible to the core study team as well as the head of research management for auditing purposes. Personal information is password-protected and stored in a secure electronic vault, separately from outcome data. All data will be stored for 15 years after the termination of the study. The research management of the Faculty of Human Sciences of the University of Bern supervises data collection and storage in quarterly meetings.

All participants create password-protected accounts for the online intervention and are allocated a unique identification number. The online intervention is hosted on a secure platform of the University of Bern. All questionnaire and interview data are recorded via Qualtrics [64]. Qualtrics uses the same unique identification number. Thus, no personal data are entered in Qualtrics. EMA data are assessed with the SEMA^3^ app and saved on a server of the University of Melbourne [65]. SEMA^3^ requires personal data only to send out invitations and deletes them immediately afterward. For identification, participants are allocated a SEMA^3^ numeric identification code.

### Plan of Analysis

All analyses will follow the intention-to-treat approach. Patterns of missing data in the Growth Mixture Models will be analyzed, and in case of “missing at random” or “missing completely at random,” multiple imputation will be applied.

#### Main Analyses

The primary outcome, that is, change in social anxiety, will be tested using Linear Mixed Effects Models. Time (baseline, midintervention, post intervention, and follow-up), intervention condition (SOPHIE and CAU), diagnostic status (SAD and subclinical social anxiety), and interactions (time*intervention condition; time*diagnostic status; intervention condition*diagnostic status; time*intervention condition*diagnostic status) will be specified as fixed effects, baseline SPIN value will be added as a fixed covariate and participant as a random effect to allow for between-person variation.

#### Further Analyses

Secondary outcomes (ie, global functioning, general anxiety symptoms, depressive symptoms, quality of life, self-esteem, and guardians’ rating of their child’s social anxiety) will be analyzed using Linear Mixed Effects Model as described earlier. In further analyses, therapy expectancy, therapy motivation (both adolescents’ and guardians’), and satisfaction with the intervention will be added as moderators. Categorical outcomes (eg, diagnostic status) will be analyzed using General Linear Mixed Effect Models with intervention condition, diagnostic status, and time course as fixed effects and participants as random effect. Additionally, Growth Mixture Modeling will be used to identify subpopulations with comparable growth trajectories of social anxiety and secondary outcomes over time (latent classes) and to identify predictors of these classes.

#### Mediation Analyses

Mediation effects of therapeutic alliance and adherence to the intervention will be analyzed in a 1-1-1 multilevel mediation model with all variables measured at assessment time points (ie, baseline, midintervention, and post intervention), and all causal paths allowed to vary between participants.

Mediation effects of mechanisms of change in social anxiety will be explored in 2 different lower-level mediation analyses using multilevel modeling. In a 2-1-1 mediation analysis, time will be included as a fixed effect, baseline social anxiety as a level-2 fixed effect predictor, mechanisms of change (ie, automatic thoughts, avoidance, self-focused attention, pre-event, and postevent processing) as a level-1 random effect mediator, and momentary social anxiety as a level-1 random effect outcome. In a 1-1-1 mediation analysis, all constructs will be assessed at level-1. Thereby, social anxiety as a random effect predictor will be assessed concurrent with the random effect mediator, mechanisms of change in social anxiety, and the random effect outcome social anxiety will be assessed 1 prompt later (lead analysis).

#### Network Analyses

Baseline, mid-, post-, and follow-up data of current levels of symptoms and functioning will be modeled by network analysis to explore how networks differ by age, gender, diagnostic status, intervention condition, and assessment point in terms of centrality, closeness, betweenness, and clustering.

#### Sample Size

The sample size of 222 was determined in 2 a priori power analyses for the primary research question using G*Power [106] based on an intention-to-treat approach to account for possible dropouts and repeated measures ANOVA. In the SAD group, we expected a small to moderate effect size (Cohen *d*=0.35) [18,107], and for the group with subclinical anxiety, a small effect size (Cohen *d*=0.20) [38,108-110]. For both groups, we assumed an α level of 5%, power of 80%, and a correlation between measurements of *r*=0.4. Consequently, the SAD group should consist of 56 participants, and the subclinical social anxiety group of 166 participants.

## Results

Data collection started in August 2021, and as of April 2023, a total of 106 participants are enrolled in the study. First results are expected in 2024.

## Discussion

Due to the limited availability of mental health services for adolescents, online interventions offer the opportunity to provide early, low-threshold, and low-cost help. In this protocol, we describe an RCT that aims to test the efficacy of an online intervention for adolescents with subclinical social anxiety or a diagnosis of SAD compared to a CAU control group. A positive evaluation of this intervention holds several implications for the future: first, it may offer an efficacious intervention for both clinical as well as subclinical levels of social anxiety; second, the delivery format provides a high level of confidentiality as well as autonomy and may reach more adolescents in need than face-to-face psychotherapy interventions, in particular, those who need care but have not sought help yet; and third, it could also precede or complement more resource-intensive face-to-face interventions in terms of a stepped care approach [111-113].

Previous studies have provided initial evidence for the efficacy of online interventions for adolescents with social anxiety [28-30]. The study described in this protocol may extend them in several ways. So far, a comprehensive understanding on empirical evidence of age-adapted treatments to meet the needs of developing adolescents has yet to be fully established [47]. In this regard, findings based on moderator analyses of intervention effects and network analysis may provide more profound insights into which subgroups of adolescents an online intervention are especially beneficial and for which a different delivery format might be indicated [16]. Next, the empirical investigation of conceptually derived mechanisms of change may complement the efficacy and moderator analyses by highlighting which factors are particularly important for clinical change [114,115]. Lastly, to the best of our knowledge, this would be the first study to provide information on whether the same online intervention can be used as both indicated prevention and therapy for adolescents with social anxiety.

Despite these promises, this study will have some limitations. The self-selected sample will limit the generalizability of the study. Although the first version of the SOPHIE program was well accepted by adolescents, it is unlikely that 1 intervention is suitable for all participants spanning a large age range associated with heterogeneous needs and capacities. In this aspect, results on efficacy among subgroups that benefit most from the intervention, for example, based on their age, gender, or symptom severity, or based on idiographic analyses of the data, will be beneficial to better personalize interventions in the future [116]. Finally, there is some evidence that even answering EMA questions may lead to a change in behavior [117]. Since both the intervention and control group receive EMA prompts, this may have an impact on between-group comparisons.

Notwithstanding these limitations, the findings of this RCT may lead to a better understanding of SAD in adolescents, may provide important insights into the subgroups of adolescents for whom an online intervention may be an important complementary mental health service, and may help to optimize the effects of current interventions for SAD and subclinical social anxiety in adolescents by targeting the identified mechanism of change more directly. Overall, this online intervention may thereby offer a low-threshold prevention and treatment option to reduce the treatment gap and improve mental health care in adolescents.

## Abbreviations

CAU: care-as-usual
CBT: cognitive behavioral therapy
EMA: ecological momentary assessments
RCT: randomized controlled trial
SAD: social anxiety disorder
SEMA3: Smartphone Ecological Momentary Assessment
SPIN: Social Phobia Inventory

## Data Availability

Data sharing is not applicable to this paper as no data sets were generated or analyzed for this protocol.
